# Supplementary figures and images for: PIWI-interacting RNAs are differentially expressed during cardiac differentiation of human pluripotent stem cells
Source: PLoS One. 2020 May 5;15(5):e0232715. doi: 10.1371/journal.pone.0232715 (PMC7199965; doi:10.1371/journal.pone.0232715)

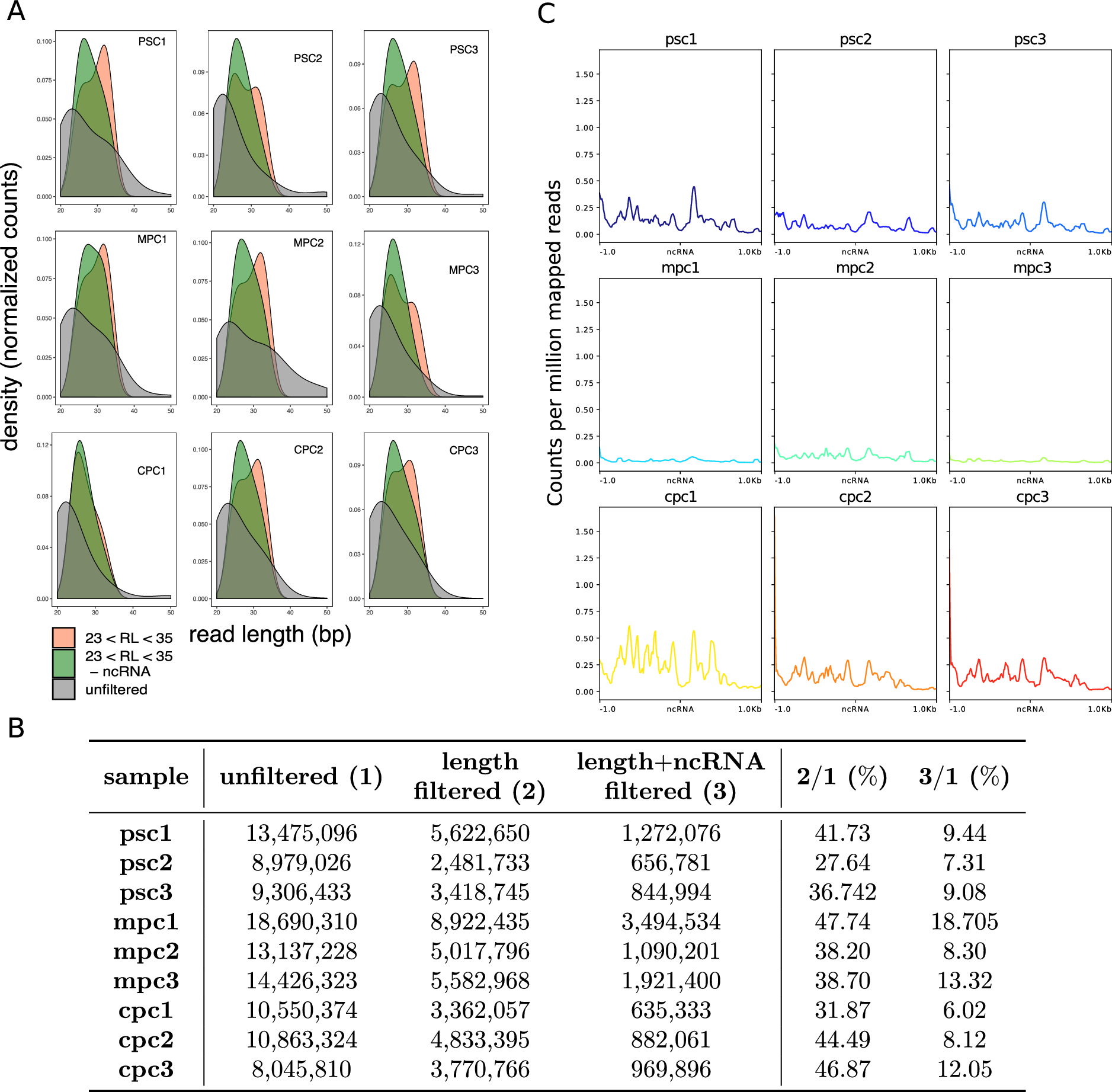

Supplement: S1 Fig — Related to Fig 1. A) Read length expressed as a function of density for pluripotent (PSC1, PSC2 and PSC3), mesoderm progenitor (MPC1, MPC2 and MPC3) and cardiomyocytes (CPC1, CPC2 and CPC3) samples before filtering (unfiltered) and after filtering (23 < RL < 35 and -ncRNA). Color key is indicated in the plot. B) Number of mapped reads. Reads were counted before processing (1) and after being filtered by length (2) and other ncRNAs (3). Remaining reads after processing are expressed as percentage(%) of unfiltered reads (2/1 and 3/1). C) Analysis of coverage on non coding RNAs loci from DASHR database for fully processed normalized (counts per million) samples. (TIF) [file pone.0232715.s001.tif]

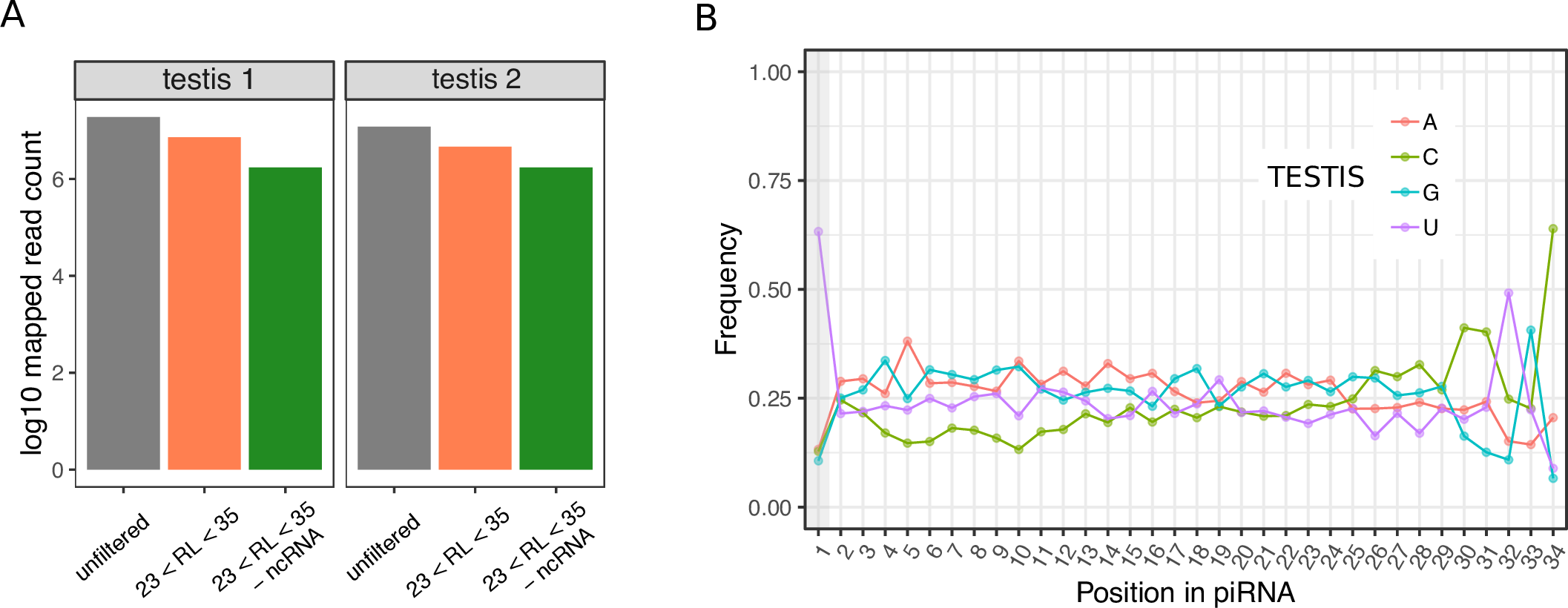

Supplement: S2 Fig — Related to Fig 2. A) Number of mapped reads after employing the pipeline described in Fig 1 in human testis samples downloaded from ENCODE (merged replicates). B) Frequency of bases per position in processed mapped reads. (TIF) [file pone.0232715.s002.tif]

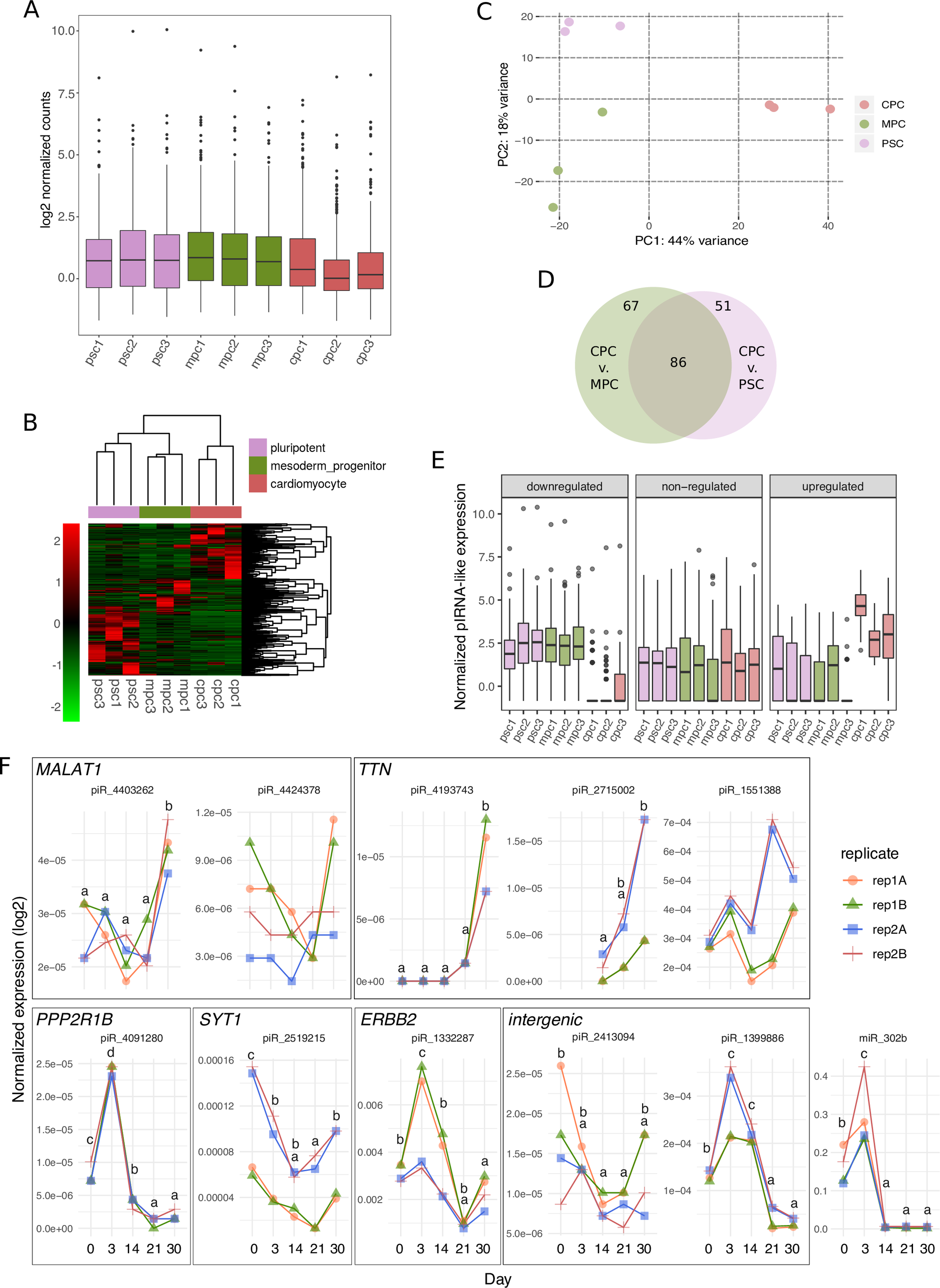

Supplement: S3 Fig — Related to Fig 3. A) Boxplot showing reads for the nine samples normalized by library depth and expressed as log2 counts per million (CPM). B) Heatmap in log2 CPM of piRNA transcripts from a. Hard unsupervised clustering was performed on rows (piRNA ID) and columns (sample ID), and is shown as dendrograms. Color keys for heatmap and phenotype are indicated to left and in the top right corner of the graph, respectively. C) Principal Component Analysis performed on DESeq2 normalized counts. The color key is indicated to the right of the plot. D) Overlap of differentially expressed piRNA transcripts in CPC versus MPC (153; green circle) and PSC (137; purple circle). E) Normalized expression of piRNA transcripts upregulated, dowregulated and non-regulated with respect to CPC. F) Ten piRNA transcripts were evaluated by qPCR using a specific retrotranscription protocol designed for small RNAs in day 0, 3, 14, 21 and 30 of cardiac differentiation. piR-4403262 and piR-4424378 originate from MALAT1; piR-4193743, piR-2715002 and piR-1551388 are produced from TTN; piR-4091280 from PPP2R1B; piR-2519215 from SYT1; piR-1332287 from ERBB2; piR-2413094 and piR-1399886 from intergenic regions. Expression of mir-302b -marker of pluripotency- was analyzed to assess protocol success. Results of four different RT reactions (rep1.A, rep1.B, rep2.A and rep2.B) from two independent experiments (rep1 and rep2) are shown after normalization by small RNA RNU6B. Statistically significant differences are displayed as letters in the graphs (p<0.05). (TIF) [file pone.0232715.s003.tif]

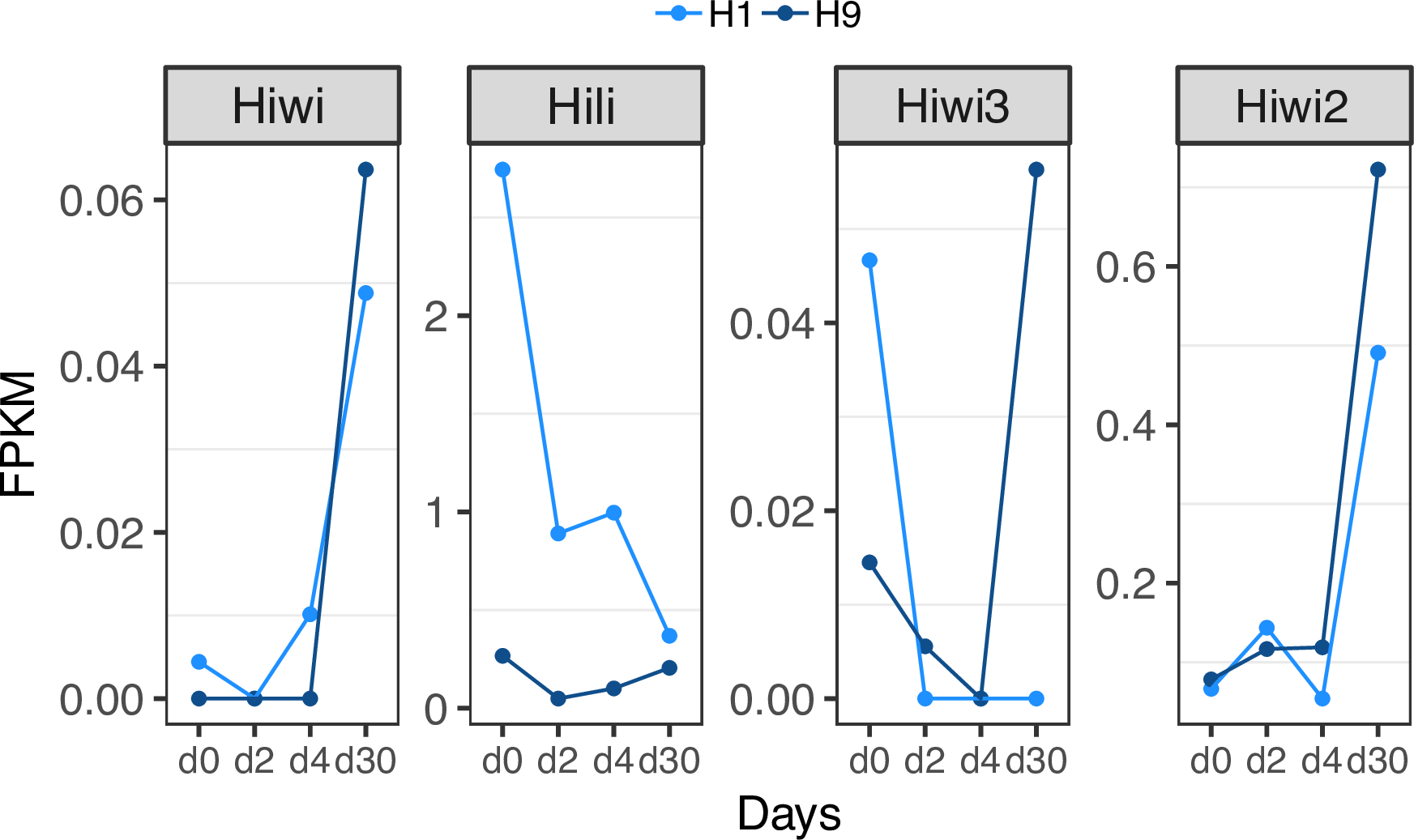

Supplement: S4 Fig — Related to Fig 4. Normalized RNA-seq counts (FPKM) from H1 and H9 cell lines were downloaded from GEO. (TIF) [file pone.0232715.s004.tif]

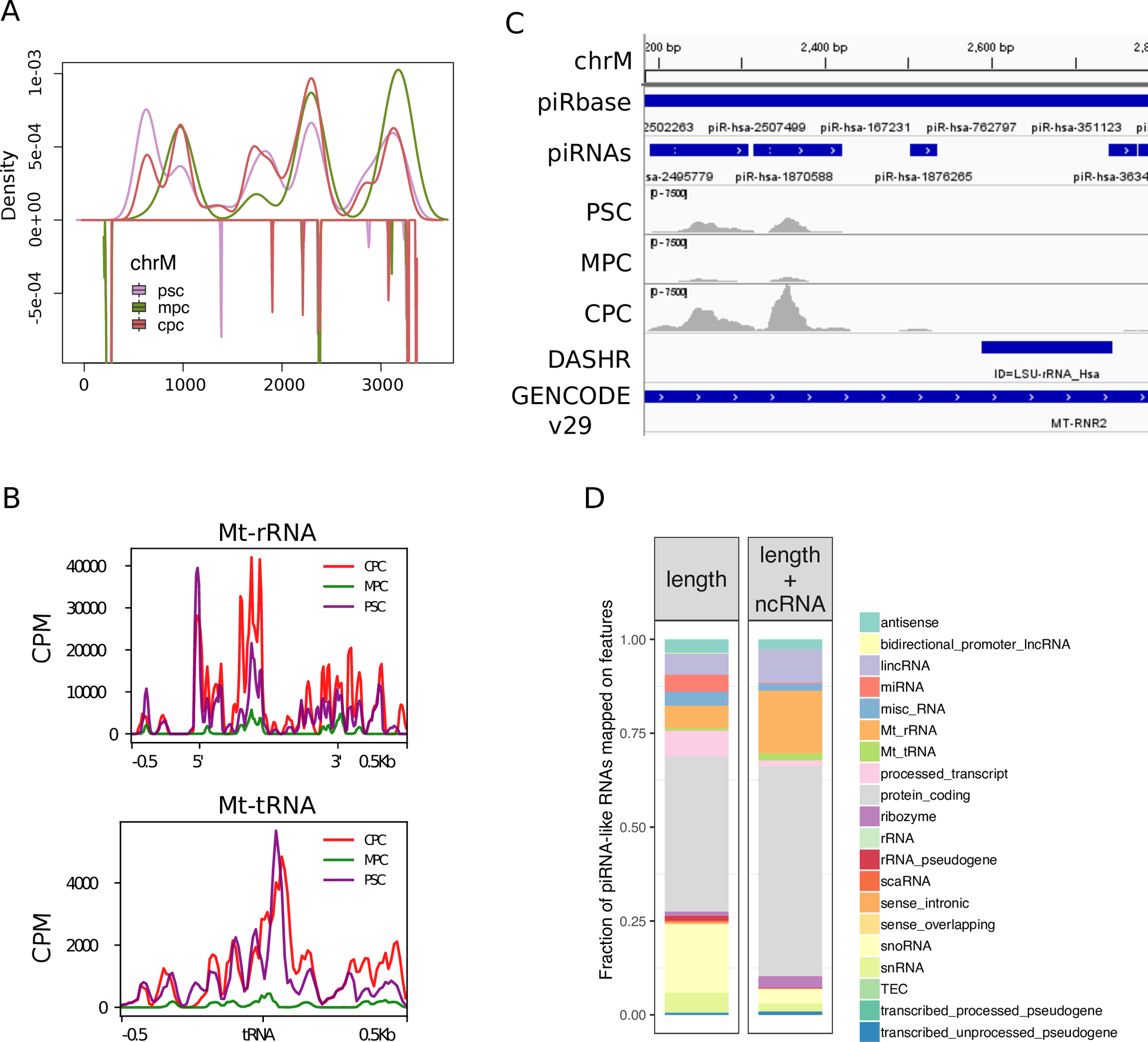

Supplement: S5 Fig — Related to Fig 5. A) Distribution of PSC, MPC and CPC mapped reads (merged replicates) as a function of density over a fraction of mitochondrial chromosome (chrM:1–4000). Profiles above zero correspond to plus strand and below zero to the minus strand. Color key is located at the bottom left corner of the plot. B) Coverage profiles in counts per million mapped reads (CPM) on the entire mitochondrial rRNA extension (MT-rRNA) and center of tRNA (MT-tRNA). Direction of rRNA genes are indicated by 5‘ and 3‘. C) Image captured from IGV software over a portion of the human mitochondrial chromosome (chrM:2,184–2,780). The tracks from top to bottom are: piRbase annotated piRNAs (piRbase), piRNAs identified in our samples (piRNAs), coverage profiles of PSC, MPC and CPC, DASHR database ncRNA annotations (DASHR) and GENCODE v29 gene annotations (GENCODE v29). D) Fraction of piRNA transcripts mapped to genomic features annotated in GENCODE v29 database in length-filtered samples (length) compared to length+ncRNA-filtered (length+ncRNA) samples. Color key for features is indicated to the right of the bars. (TIF) [file pone.0232715.s005.tif]

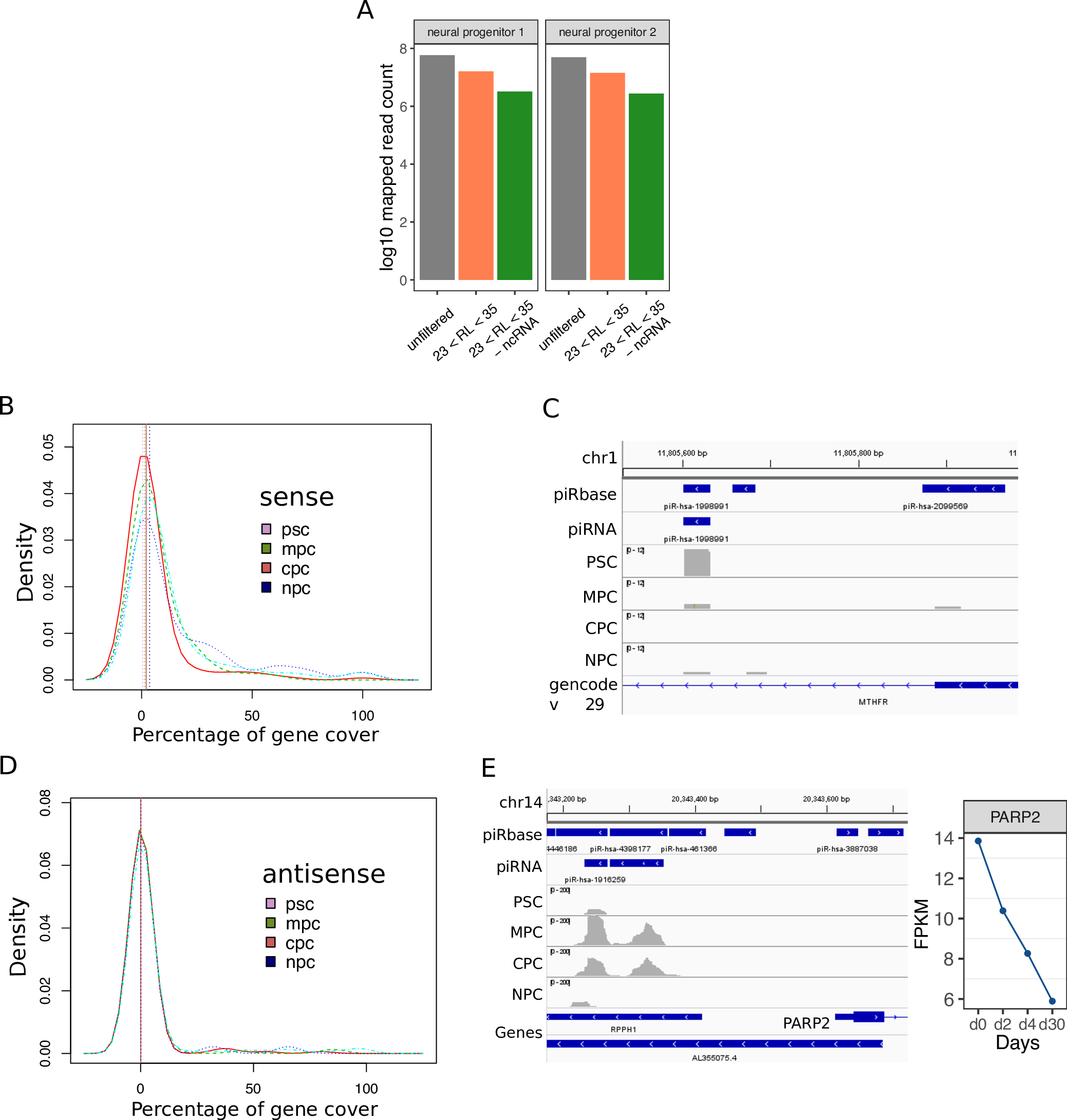

Supplement: S6 Fig — Related to Figs 6 and 7. A) NPC sample processing. Number of mapped reads after employing the pipeline described in N in neural progenitor samples downloaded from ENCODE project. B) Density estimation of percent (%) coverage on protein coding genes intersected by piRNA transcripts in sense orientation. Vertical lines indicate medians of each curve. C) Image captured from IGV software portraying mapped reads of PSC, MPC, CPC and NPC samples on identified piRNA RNAs. Tracks for piRNA annotation database (piRbase) and gene features (GENCODE v29) are shown. D) Density estimation as in a, in antisense orientation. E) Left panel shows IGV capture depicting piRNAs in PARP2 vecinity. Expression dynamic of PARP2 gene in RNA-seq samples from H9 cells differentiated to CM is shown to the right. (TIF) [file pone.0232715.s006.tif]
