# Supplementary material for: PIWI-interacting RNAs are differentially expressed during cardiac differentiation of human pluripotent stem cells
Source: PLoS One. 2020 May 5;15(5):e0232715. doi: 10.1371/journal.pone.0232715 (PMC7199965; doi:10.1371/journal.pone.0232715)
Supplement: S1 Table — Transcripts are arranged in descending order of expression (row mean). Differentially regulated piRNA transcripts in CPC are indicated in light blue shading (downregulated) and light red shading (upregulated). (PDF) [file pone.0232715.s007.pdf]

	piRNA	psc1	psc2	psc3	mpc1	mpc2	mpc3	cpc1	cpc2	cpc3
1	piR-hsa-1389062	252.26	1263.26	1335.60	666.72	757.88	83.67	110.68	260.73	280.30
2	piR-hsa-4381848_2	86.38	70.78	77.08	78.63	235.99	77.84	81.81	31.18	79.49
3	piR-hsa-3732777	17.49	35.39	110.80	105.40	106.53	60.32	24.06	55.58	73.21
4	piR-hsa-1904126	12.72	40.01	49.38	96.20	72.82	83.67	57.75	39.76	89.25
5	piR-hsa-1919272	50.88	26.16	33.72	10.87	72.82	3.89	178.05	41.12	60.66
6	piR-hsa-3660133	88.50	67.70	77.08	44.34	64.73	35.03	1.60	0.00	0.00
7	piR-hsa-3817390	25.97	69.24	49.38	23.42	31.02	60.32	0.00	1.36	2.79
8	piR-hsa-151466	25.44	15.39	26.50	18.40	13.49	0.00	44.91	16.27	77.40
9	piR-hsa-2539762	20.14	9.23	21.68	2.51	18.88	1.95	81.81	22.14	41.14
10	piR-hsa-4091280	22.79	112.32	28.90	5.02	6.74	25.30	0.00	0.00	0.70
11	piR-hsa-1939085	0.00	0.00	0.00	148.90	2.70	3.89	33.69	6.78	2.09
12	piR-hsa-169217	49.29	26.16	56.60	19.24	29.67	9.73	0.00	0.90	2.09
13	piR-hsa-97458	0.00	3.08	3.61	22.59	57.99	93.40	0.00	0.00	0.00
14	piR-hsa-665910	1.06	0.00	1.20	2.51	0.00	7.78	14.44	14.01	131.08
15	piR-hsa-2346976	19.61	60.01	30.11	22.59	22.93	9.73	0.00	0.00	0.70
16	piR-hsa-1933276	2.65	0.00	3.61	107.08	36.41	13.62	0.00	0.45	1.39
17	piR-hsa-3634065	5.30	6.15	8.43	0.00	10.79	0.00	105.87	6.78	19.52
18	piR-hsa-2489909	0.00	3.08	3.61	6.69	5.39	114.81	4.81	3.61	7.67
19	piR-hsa-3658275	14.84	7.69	3.61	1.67	16.18	3.89	67.37	14.91	18.83
20	piR-hsa-2832647	7.42	46.16	6.02	7.53	2.70	27.24	20.85	14.91	5.58
21	piR-hsa-3352181	12.72	16.93	10.84	89.51	2.70	1.95	0.00	0.00	2.09
22	piR-hsa-151249	7.95	10.77	19.27	40.15	16.18	15.57	1.60	18.98	4.18
23	piR-hsa-316012	4.77	13.85	6.02	2.51	10.79	31.13	20.85	14.46	26.50
24	piR-hsa-2479371	21.73	10.77	14.45	5.02	24.27	1.95	30.48	8.59	10.46
25	piR-hsa-611204	6.89	0.00	8.43	51.87	45.85	9.73	1.60	0.00	1.39
26	piR-hsa-2513278	10.07	9.23	19.27	27.61	39.11	13.62	3.21	0.00	1.39
27	piR-hsa-3974794	32.86	18.46	21.68	15.89	6.74	15.57	3.21	1.81	4.88
28	piR-hsa-2780538	15.37	35.39	24.09	7.53	9.44	25.30	0.00	0.90	2.79
29	piR-hsa-1872085_4	4.24	26.16	8.43	25.10	4.05	31.13	3.21	5.87	8.37
30	piR-hsa-2526525	6.36	4.62	6.02	0.00	5.39	0.00	73.79	2.26	11.16
31	piR-hsa-745484	2.12	3.08	0.00	62.74	2.70	0.00	33.69	3.61	0.70
32	piR-hsa-147461	5.83	3.08	6.02	32.62	18.88	29.19	1.60	7.68	0.70
33	piR-hsa-4460706	11.66	4.62	1.20	0.00	10.79	1.95	41.71	15.36	16.73
34	piR-hsa-1927965	11.13	12.31	3.61	0.00	10.79	0.00	51.33	2.26	9.76
35	piR-hsa-4303719	13.78	24.62	36.13	14.22	2.70	9.73	0.00	0.00	0.00
36	piR-hsa-114666	18.02	15.39	26.50	1.67	9.44	29.19	0.00	0.45	0.00
37	piR-hsa-1870588	18.02	1.54	9.63	1.67	16.18	0.00	35.29	9.49	8.37
38	piR-hsa-7760463	1.59	0.00	0.00	33.46	55.29	3.89	1.60	0.00	4.18
39	piR-hsa-1706026	1.06	4.62	0.00	74.45	9.44	0.00	8.02	0.00	1.39
40	piR-hsa-2856604	4.24	23.08	24.09	25.93	9.44	7.78	0.00	0.45	2.09
41	piR-hsa-1233052	17.49	3.08	7.23	0.00	12.14	0.00	41.71	9.04	4.18
42	piR-hsa-2482189	2.12	10.77	0.00	49.36	6.74	3.89	14.44	3.16	3.49
43	piR-hsa-2519215	15.90	18.46	37.33	7.53	5.39	7.78	0.00	0.00	0.00
44	piR-hsa-2863156	5.30	15.39	6.02	20.08	4.05	35.03	4.81	0.90	0.70
45	piR-hsa-151136	8.48	21.54	14.45	16.73	5.39	11.68	3.21	8.13	0.70
46	piR-hsa-214132	12.72	23.08	19.27	8.37	12.14	13.62	0.00	0.45	0.00
47	piR-hsa-1332287	6.89	52.32	13.25	0.00	5.39	5.84	0.00	2.26	1.39
48	piR-hsa-1923208	6.89	7.69	2.41	3.35	2.70	1.95	35.29	5.87	19.52
49	piR-hsa-1463989	2.65	21.54	27.70	6.69	5.39	19.46	0.00	0.00	0.00
50	piR-hsa-2413094	12.72	30.77	20.47	0.84	10.79	7.78	0.00	0.00	0.00
51	piR-hsa-2299252	14.84	4.62	28.90	10.04	8.09	15.57	0.00	0.00	0.00
52	piR-hsa-2831593	6.89	1.54	27.70	11.71	14.83	1.95	0.00	11.75	5.58
53	piR-hsa-1429070	2.12	4.62	7.23	3.35	6.74	56.43	0.00	0.00	0.00
54	piR-hsa-2268195	14.84	4.62	32.52	1.67	4.05	13.62	1.60	1.36	4.18
55	piR-hsa-4020841	5.83	23.08	30.11	1.67	5.39	11.68	0.00	0.00	0.00
56	piR-hsa-1647694	9.54	12.31	30.11	8.37	6.74	7.78	0.00	0.00	1.39
57	piR-hsa-359160_2	2.12	7.69	3.61	25.10	8.09	9.73	1.60	8.59	9.06
58	piR-hsa-1001021	4.77	4.62	1.20	23.42	6.74	17.51	11.23	0.00	5.58
59	piR-hsa-772699	0.00	0.00	0.00	53.54	8.09	7.78	3.21	0.90	1.39

	piRNA	psc1	psc2	psc3	mpc1	mpc2	mpc3	cpc1	cpc2	cpc3
60	piR-hsa-3916570	15.90	7.69	28.90	11.71	6.74	3.89	0.00	0.00	0.00
61	piR-hsa-1916259	3.18	3.08	1.20	23.42	8.09	3.89	25.67	4.52	0.70
62	piR-hsa-4322932	6.89	24.62	28.90	4.18	4.05	3.89	0.00	0.45	0.70
63	piR-hsa-2521457	3.71	4.62	3.61	1.67	4.05	0.00	41.71	5.42	6.97
64	piR-hsa-1903779	1.59	3.08	7.23	4.18	6.74	42.81	4.81	0.00	0.70
65	piR-hsa-2646470	0.53	4.62	8.43	22.59	21.58	3.89	1.60	2.26	4.88
66	piR-hsa-2497478	24.38	4.62	8.43	1.67	5.39	0.00	19.25	2.26	3.49
67	piR-hsa-1875212	4.24	3.08	1.20	0.00	4.05	0.00	38.50	8.13	9.76
68	piR-hsa-4387218	0.00	0.00	0.00	0.84	1.35	0.00	4.81	27.11	31.38
69	piR-hsa-6913457	10.60	16.93	7.23	20.91	5.39	1.95	1.60	0.00	0.00
70	piR-hsa-1756618	2.65	0.00	3.61	19.24	21.58	3.89	9.62	0.45	3.49
71	piR-hsa-2592846	0.00	0.00	0.00	0.00	0.00	0.00	60.96	0.90	2.09
72	piR-hsa-1908839	6.36	0.00	2.41	1.67	17.53	0.00	16.04	10.84	7.67
73	piR-hsa-1576285	0.00	3.08	0.00	1.67	1.35	13.62	3.21	20.33	18.83
74	piR-hsa-1862211	0.00	7.69	0.00	12.55	0.00	21.40	11.23	3.61	2.79
75	piR-hsa-3658742	6.89	1.54	4.82	0.00	24.27	0.00	16.04	2.26	2.79
76	piR-hsa-1872235	6.89	3.08	9.63	0.00	8.09	1.95	16.04	7.23	4.88
77	piR-hsa-1277994	3.71	0.00	8.43	2.51	29.67	1.95	4.81	0.00	6.28
78	piR-hsa-343382	4.24	4.62	2.41	1.67	4.05	1.95	19.25	9.04	9.76
79	piR-hsa-2027369	14.84	16.93	12.04	4.18	2.70	5.84	0.00	0.00	0.00
80	piR-hsa-1399886	2.12	24.62	4.82	15.89	8.09	0.00	0.00	0.00	0.00
81	piR-hsa-1798104	7.42	15.39	24.09	2.51	1.35	3.89	0.00	0.00	0.00
82	piR-hsa-1875155	2.12	21.54	21.68	3.35	0.00	5.84	0.00	0.00	0.00
83	piR-hsa-1548068	1.59	6.15	13.25	4.18	10.79	0.00	1.60	8.13	8.37
84	piR-hsa-721859_9	2.12	9.23	8.43	6.69	4.05	17.51	3.21	0.90	1.39
85	piR-hsa-3732088	16.43	3.08	20.47	1.67	1.35	1.95	0.00	3.16	4.88
86	piR-hsa-1303811	1.59	4.62	0.00	31.79	1.35	3.89	9.62	0.00	0.00
87	piR-hsa-2481097	6.89	3.08	6.02	0.00	12.14	0.00	19.25	4.07	1.39
88	piR-hsa-4028152	2.12	15.39	0.00	5.02	2.70	25.30	1.60	0.00	0.70
89	piR-hsa-389007	2.65	4.62	2.41	9.20	10.79	19.46	3.21	0.00	0.00
90	piR-hsa-2477264	5.30	6.15	6.02	0.00	10.79	0.00	17.64	4.07	2.09
91	piR-hsa-255984	2.12	3.08	12.04	5.86	2.70	23.35	0.00	0.00	2.09
92	piR-hsa-137136	7.42	7.69	9.63	8.37	8.09	9.73	0.00	0.00	0.00
93	piR-hsa-3807498	7.95	13.85	15.66	1.67	6.74	3.89	0.00	0.00	0.70
94	piR-hsa-783698	15.37	7.69	6.02	5.02	13.49	1.95	0.00	0.00	0.00
95	piR-hsa-1988800	7.95	4.62	12.04	3.35	14.83	5.84	0.00	0.00	0.00
96	piR-hsa-1941780	3.71	6.15	3.61	1.67	2.70	1.95	19.25	1.36	7.67
97	piR-hsa-3875265	10.60	9.23	10.84	5.86	9.44	1.95	0.00	0.00	0.00
98	piR-hsa-1876265	4.24	4.62	3.61	0.00	0.00	0.00	19.25	6.33	9.76
99	piR-hsa-368987	6.36	4.62	4.82	5.02	4.05	3.89	9.62	5.87	3.49
100	piR-hsa-1890632	6.36	4.62	7.23	0.00	5.39	0.00	11.23	4.52	8.37
101	piR-hsa-4078407	2.12	20.00	9.63	10.04	1.35	3.89	0.00	0.00	0.00
102	piR-hsa-76848	4.24	4.62	1.20	1.67	1.35	25.30	1.60	1.36	5.58
103	piR-hsa-2565910	0.53	12.31	4.82	5.02	5.39	15.57	0.00	1.81	1.39
104	piR-hsa-1205256	3.18	4.62	1.20	21.75	0.00	11.68	3.21	0.45	0.00
105	piR-hsa-2840936	10.60	0.00	1.20	0.00	6.74	0.00	19.25	4.97	2.79
106	piR-hsa-20628	3.71	9.23	12.04	2.51	8.09	9.73	0.00	0.00	0.00
107	piR-hsa-753191	0.00	0.00	0.00	0.00	0.00	0.00	35.29	5.42	4.18
108	piR-hsa-3683883	2.12	3.08	6.02	20.08	1.35	1.95	6.42	0.00	2.79
109	piR-hsa-1690788	1.59	4.62	4.82	5.86	6.74	17.51	0.00	0.00	2.09
110	piR-hsa-2530015	6.89	3.08	1.20	2.51	0.00	0.00	16.04	2.26	11.16
111	piR-hsa-346271	3.71	3.08	15.66	1.67	2.70	0.00	3.21	1.81	10.46
112	piR-hsa-3546008	6.89	3.08	8.43	5.02	14.83	3.89	0.00	0.00	0.00
113	piR-hsa-5982715	7.42	13.85	2.41	6.69	2.70	7.78	0.00	0.90	0.00
114	piR-hsa-2398570	1.06	6.15	3.61	2.51	12.14	15.57	0.00	0.00	0.70
115	piR-hsa-1883972	6.36	0.00	0.00	0.00	2.70	0.00	19.25	3.61	9.76
116	piR-hsa-3718263_3	0.00	0.00	0.00	0.00	0.00	0.00	33.69	5.87	2.09
117	piR-hsa-4472891	2.12	3.08	9.63	11.71	6.74	5.84	0.00	1.81	0.70
118	piR-hsa-2208850	4.24	15.39	7.23	5.02	1.35	7.78	0.00	0.00	0.00

	piRNA	psc1	psc2	psc3	mpc1	mpc2	mpc3	cpc1	cpc2	cpc3
119	piR-hsa-785969	2.12	3.08	0.00	6.69	0.00	1.95	24.06	1.36	1.39
120	piR-hsa-163695	6.36	10.77	7.23	0.84	6.74	7.78	0.00	0.00	0.70
121	piR-hsa-2490897	6.89	0.00	1.20	1.67	0.00	0.00	11.23	5.42	13.95
122	piR-hsa-2151268	1.59	7.69	3.61	7.53	8.09	11.68	0.00	0.00	0.00
123	piR-hsa-771714_3	3.71	4.62	2.41	0.00	0.00	3.89	17.64	0.90	6.97
124	piR-hsa-1920687	4.24	4.62	1.20	1.67	4.05	0.00	12.83	5.42	5.58
125	piR-hsa-2351941	5.30	23.08	2.41	2.51	2.70	1.95	1.60	0.00	0.00
126	piR-hsa-3265318	3.18	13.85	6.02	1.67	2.70	11.68	0.00	0.00	0.00
127	piR-hsa-2426792	2.12	24.62	4.82	6.69	0.00	0.00	0.00	0.00	0.70
128	piR-hsa-2503702	13.25	0.00	8.43	0.00	4.05	0.00	6.42	3.16	2.79
129	piR-hsa-161264_3	1.06	0.00	1.20	8.37	5.39	21.40	0.00	0.00	0.00
130	piR-hsa-1912443	1.59	0.00	1.20	0.00	1.35	0.00	28.87	2.26	2.09
131	piR-hsa-2230204	8.48	6.15	15.66	3.35	2.70	0.00	0.00	0.00	0.70
132	piR-hsa-2090264	7.42	1.54	25.29	0.84	1.35	0.00	0.00	0.00	0.00
133	piR-hsa-1242358	0.00	0.00	0.00	0.00	0.00	0.00	32.08	2.26	2.09
134	piR-hsa-724912	2.65	1.54	3.61	22.59	1.35	0.00	3.21	0.45	0.70
135	piR-hsa-7765828	1.06	7.69	3.61	13.38	4.05	5.84	0.00	0.00	0.00
136	piR-hsa-2172087	1.59	10.77	3.61	5.02	2.70	7.78	3.21	0.90	0.00
137	piR-hsa-1425899	0.00	4.62	1.20	6.69	2.70	17.51	0.00	1.36	1.39
138	piR-hsa-1921188	1.59	3.08	1.20	0.00	10.79	0.00	14.44	3.61	0.70
139	piR-hsa-1917139	2.65	1.54	1.20	0.00	4.05	0.00	20.85	1.36	3.49
140	piR-hsa-3829948	0.53	3.08	0.00	13.38	4.05	13.62	0.00	0.00	0.00
141	piR-hsa-3735787	7.42	3.08	2.41	0.00	2.70	0.00	12.83	4.07	2.09
142	piR-hsa-5996985	0.00	0.00	0.00	0.00	0.00	0.00	28.87	5.42	0.00
143	piR-hsa-2989729	6.36	6.15	13.25	0.84	2.70	3.89	0.00	0.00	0.00
144	piR-hsa-21839	0.53	3.08	0.00	24.26	2.70	1.95	0.00	0.45	0.00
145	piR-hsa-1872463	0.00	4.62	1.20	1.67	0.00	19.46	0.00	3.16	2.79
146	piR-hsa-508592	2.12	13.85	7.23	2.51	1.35	5.84	0.00	0.00	0.00
147	piR-hsa-2252211	1.59	6.15	3.61	0.84	20.23	0.00	0.00	0.45	0.00
148	piR-hsa-4110708	2.65	27.70	1.20	0.84	0.00	0.00	0.00	0.00	0.00
149	piR-hsa-298158	4.24	6.15	6.02	7.53	4.05	3.89	0.00	0.00	0.00
150	piR-hsa-7892960	6.36	1.54	3.61	4.18	2.70	1.95	3.21	4.52	3.49
151	piR-hsa-2395910	2.65	3.08	3.61	3.35	1.35	17.51	0.00	0.00	0.00
152	piR-hsa-2536290	0.53	12.31	3.61	8.37	1.35	3.89	0.00	0.45	0.70
153	piR-hsa-2152778	2.65	10.77	6.02	5.02	2.70	3.89	0.00	0.00	0.00
154	piR-hsa-5077723	0.53	4.62	12.04	8.37	5.39	0.00	0.00	0.00	0.00
155	piR-hsa-1291516	7.42	6.15	10.84	2.51	1.35	0.00	0.00	0.90	1.39
156	piR-hsa-1901970	12.19	7.69	0.00	3.35	5.39	1.95	0.00	0.00	0.00
157	piR-hsa-2450089_2	0.00	1.54	4.82	3.35	1.35	19.46	0.00	0.00	0.00
158	piR-hsa-108574	3.71	7.69	4.82	3.35	8.09	1.95	0.00	0.00	0.70
159	piR-hsa-1259653	2.65	1.54	2.41	0.84	1.35	1.95	16.04	1.81	0.70
160	piR-hsa-3136454	2.65	16.93	2.41	1.67	2.70	1.95	0.00	0.00	0.70
161	piR-hsa-2286229	4.24	3.08	9.63	4.18	5.39	1.95	0.00	0.45	0.00
162	piR-hsa-2209630	2.65	0.00	0.00	23.42	2.70	0.00	0.00	0.00	0.00
163	piR-hsa-1696540	0.00	0.00	0.00	0.00	0.00	1.95	24.06	1.81	0.70
164	piR-hsa-6245615	3.71	4.62	7.23	0.00	10.79	1.95	0.00	0.00	0.00
165	piR-hsa-2072163	1.06	4.62	2.41	1.67	2.70	5.84	8.02	1.81	0.00
166	piR-hsa-1557538	0.00	0.00	2.41	8.37	9.44	7.78	0.00	0.00	0.00
167	piR-hsa-2844156	7.42	3.08	4.82	5.86	0.00	3.89	0.00	1.36	1.39
168	piR-hsa-6744266	0.00	1.54	1.20	5.86	0.00	5.84	9.62	2.26	1.39
169	piR-hsa-1882039	1.06	0.00	0.00	0.00	0.00	0.00	17.64	2.71	6.28
170	piR-hsa-2137611	5.30	15.39	6.02	0.84	0.00	0.00	0.00	0.00	0.00
171	piR-hsa-58291	3.71	6.15	8.43	0.84	4.05	3.89	0.00	0.45	0.00
172	piR-hsa-4408495	0.00	0.00	2.41	5.86	5.39	11.68	1.60	0.45	0.00
173	piR-hsa-374600	1.06	0.00	1.20	10.87	1.35	0.00	12.83	0.00	0.00
174	piR-hsa-2829712	3.71	0.00	1.20	0.00	5.39	0.00	9.62	3.16	4.18
175	piR-hsa-3177742	3.18	4.62	7.23	1.67	0.00	9.73	0.00	0.00	0.70
176	piR-hsa-1585146	2.12	0.00	4.82	9.20	6.74	3.89	0.00	0.00	0.00
177	piR-hsa-4403577	2.65	4.62	7.23	0.84	5.39	3.89	0.00	1.36	0.70

	piRNA	psc1	psc2	psc3	mpc1	mpc2	mpc3	cpc1	cpc2	cpc3
178	piR-hsa-1528884	4.24	10.77	4.82	2.51	1.35	1.95	0.00	0.00	0.70
179	piR-hsa-2494226	4.77	3.08	0.00	0.00	2.70	0.00	8.02	2.71	4.88
180	piR-hsa-229786	0.00	4.62	0.00	2.51	2.70	15.57	0.00	0.00	0.70
181	piR-hsa-1492262	3.71	4.62	1.20	0.84	12.14	0.00	1.60	0.45	1.39
182	piR-hsa-132896	1.59	4.62	4.82	4.18	8.09	1.95	0.00	0.00	0.70
183	piR-hsa-1376916	2.12	10.77	2.41	3.35	2.70	3.89	0.00	0.45	0.00
184	piR-hsa-160969	0.53	0.00	6.02	3.35	2.70	11.68	0.00	0.00	1.39
185	piR-hsa-768321	8.48	3.08	1.20	0.84	0.00	0.00	4.81	0.90	6.28
186	piR-hsa-3842249	1.59	0.00	2.41	10.04	1.35	5.84	3.21	0.45	0.70
187	piR-hsa-362913	1.06	1.54	1.20	21.75	0.00	0.00	0.00	0.00	0.00
188	piR-hsa-1870459	3.18	0.00	4.82	0.84	6.74	0.00	4.81	2.71	2.09
189	piR-hsa-3634880	2.12	0.00	0.00	5.02	6.74	3.89	3.21	0.00	4.18
190	piR-hsa-1941637	2.65	0.00	0.00	16.73	1.35	0.00	3.21	0.45	0.70
191	piR-hsa-834074	0.00	0.00	0.00	0.00	0.00	0.00	20.85	2.71	1.39
192	piR-hsa-1900529	5.83	3.08	3.61	0.00	4.05	1.95	0.00	2.26	4.18
193	piR-hsa-2220917	0.00	3.08	1.20	11.71	8.09	0.00	0.00	0.00	0.70
194	piR-hsa-3739406	3.18	1.54	2.41	5.86	2.70	0.00	1.60	0.45	6.97
195	piR-hsa-7308134	0.00	0.00	0.00	14.22	2.70	7.78	0.00	0.00	0.00
196	piR-hsa-1708978	0.53	0.00	0.00	11.71	2.70	9.73	0.00	0.00	0.00
197	piR-hsa-3231825	3.71	12.31	3.61	1.67	1.35	1.95	0.00	0.00	0.00
198	piR-hsa-2398119	4.77	3.08	0.00	1.67	5.39	0.00	9.62	0.00	0.00
199	piR-hsa-2882083	3.71	4.62	0.00	5.86	4.05	1.95	1.60	1.36	1.39
200	piR-hsa-315964	4.77	1.54	10.84	1.67	5.39	0.00	0.00	0.00	0.00
201	piR-hsa-4030155	6.89	4.62	2.41	1.67	2.70	5.84	0.00	0.00	0.00
202	piR-hsa-2615134	3.71	3.08	2.41	10.87	4.05	0.00	0.00	0.00	0.00
203	piR-hsa-7544198	1.59	0.00	2.41	12.55	6.74	0.00	0.00	0.00	0.70
204	piR-hsa-1843231	1.06	4.62	4.82	4.18	5.39	3.89	0.00	0.00	0.00
205	piR-hsa-2670375	1.06	1.54	2.41	11.71	1.35	5.84	0.00	0.00	0.00
206	piR-hsa-2464166	1.06	3.08	0.00	0.84	1.35	17.51	0.00	0.00	0.00
207	piR-hsa-2589139	1.59	1.54	4.82	12.55	1.35	1.95	0.00	0.00	0.00
208	piR-hsa-3119265	4.24	1.54	4.82	2.51	6.74	3.89	0.00	0.00	0.00
209	piR-hsa-1531418	1.06	3.08	3.61	6.69	5.39	3.89	0.00	0.00	0.00
210	piR-hsa-2253283	2.65	4.62	0.00	11.71	2.70	1.95	0.00	0.00	0.00
211	piR-hsa-2148238	1.59	7.69	8.43	2.51	1.35	1.95	0.00	0.00	0.00
212	piR-hsa-1595580	2.12	6.15	7.23	2.51	5.39	0.00	0.00	0.00	0.00
213	piR-hsa-3978322	0.00	0.00	0.00	10.04	6.74	3.89	1.60	0.90	0.00
214	piR-hsa-1773241	2.65	1.54	3.61	3.35	8.09	3.89	0.00	0.00	0.00
215	piR-hsa-1434629	0.53	1.54	3.61	7.53	4.05	5.84	0.00	0.00	0.00
216	piR-hsa-1340768	0.53	1.54	1.20	3.35	6.74	7.78	0.00	0.45	1.39
217	piR-hsa-333507	1.59	4.62	8.43	1.67	2.70	3.89	0.00	0.00	0.00
218	piR-hsa-118348	3.18	6.15	3.61	3.35	4.05	0.00	0.00	0.45	2.09
219	piR-hsa-3623001	4.24	1.54	1.20	0.00	9.44	0.00	1.60	1.36	3.49
220	piR-hsa-2090890	2.65	4.62	4.82	4.18	2.70	3.89	0.00	0.00	0.00
221	piR-hsa-1929067	2.12	9.23	1.20	2.51	0.00	7.78	0.00	0.00	0.00
222	piR-hsa-2827579	1.06	0.00	0.00	0.00	1.35	1.95	6.42	0.90	11.16
223	piR-hsa-1707103	0.00	1.54	0.00	0.00	0.00	0.00	16.04	4.52	0.70
224	piR-hsa-1256360	0.00	0.00	0.00	9.20	2.70	9.73	0.00	0.45	0.70
225	piR-hsa-1919455	6.89	1.54	2.41	0.00	1.35	0.00	4.81	3.61	2.09
226	piR-hsa-1607096	1.06	12.31	1.20	0.84	2.70	3.89	0.00	0.00	0.70
227	piR-hsa-2515454	5.30	3.08	0.00	0.00	5.39	0.00	6.42	1.81	0.70
228	piR-hsa-368381	0.53	1.54	1.20	2.51	0.00	1.95	4.81	3.16	6.97
229	piR-hsa-4379982	4.24	4.62	1.20	0.84	9.44	0.00	1.60	0.00	0.70
230	piR-hsa-2240007	3.71	7.69	4.82	1.67	2.70	1.95	0.00	0.00	0.00
231	piR-hsa-4416099_9	0.00	0.00	1.20	0.84	0.00	19.46	0.00	0.90	0.00
232	piR-hsa-7106256	1.59	0.00	1.20	10.87	5.39	1.95	0.00	0.45	0.70
233	piR-hsa-1481120	2.12	3.08	1.20	8.37	5.39	1.95	0.00	0.00	0.00
234	piR-hsa-1284504	0.53	1.54	0.00	4.18	0.00	3.89	6.42	2.71	2.79
235	piR-hsa-2505515	0.00	0.00	0.00	7.53	6.74	7.78	0.00	0.00	0.00
236	piR-hsa-1927627	0.00	0.00	0.00	0.00	0.00	0.00	16.04	1.81	4.18

	piRNA	psc1	psc2	psc3	mpc1	mpc2	mpc3	cpc1	cpc2	cpc3
237	piR-hsa-1921551	2.12	1.54	1.20	12.55	2.70	0.00	0.00	0.45	1.39
238	piR-hsa-163499	4.77	3.08	3.61	1.67	2.70	1.95	1.60	0.45	2.09
239	piR-hsa-4202081	1.59	1.54	4.82	4.18	5.39	3.89	0.00	0.45	0.00
240	piR-hsa-147696	1.59	0.00	0.00	0.84	1.35	0.00	9.62	4.07	4.18
241	piR-hsa-2353109	0.00	12.31	1.20	0.84	1.35	5.84	0.00	0.00	0.00
242	piR-hsa-8270846	3.71	4.62	7.23	5.86	0.00	0.00	0.00	0.00	0.00
243	piR-hsa-1905680	0.53	1.54	3.61	0.84	0.00	0.00	8.02	4.07	2.79
244	piR-hsa-307961	2.12	6.15	0.00	3.35	0.00	9.73	0.00	0.00	0.00
245	piR-hsa-2333057	4.77	0.00	3.61	0.84	9.44	1.95	0.00	0.00	0.70
246	piR-hsa-1938524	0.53	0.00	1.20	0.84	1.35	1.95	11.23	1.36	2.79
247	piR-hsa-3232943	3.18	0.00	3.61	5.02	5.39	3.89	0.00	0.00	0.00
248	piR-hsa-3513154	5.30	1.54	7.23	4.18	2.70	0.00	0.00	0.00	0.00
249	piR-hsa-3674332	14.31	0.00	0.00	0.00	2.70	0.00	1.60	0.90	1.39
250	piR-hsa-2213434	6.36	3.08	7.23	4.18	0.00	0.00	0.00	0.00	0.00
251	piR-hsa-2308163	2.65	7.69	3.61	4.18	2.70	0.00	0.00	0.00	0.00
252	piR-hsa-1748898	0.00	7.69	4.82	5.02	1.35	1.95	0.00	0.00	0.00
253	piR-hsa-2525461	0.00	3.08	3.61	6.69	2.70	1.95	0.00	0.00	2.79
254	piR-hsa-1296118	3.18	0.00	2.41	0.00	2.70	0.00	8.02	3.61	0.70
255	piR-hsa-1632961	0.53	12.31	3.61	0.84	1.35	1.95	0.00	0.00	0.00
256	piR-hsa-728085	0.53	1.54	0.00	0.84	0.00	0.00	17.64	0.00	0.00
257	piR-hsa-623353	0.00	1.54	0.00	0.84	0.00	15.57	0.00	0.45	2.09
258	piR-hsa-3527815	1.06	13.85	3.61	0.00	0.00	1.95	0.00	0.00	0.00
259	piR-hsa-2478880_2	0.00	1.54	0.00	0.00	2.70	0.00	8.02	3.16	4.88
260	piR-hsa-3558751	4.24	6.15	4.82	1.67	1.35	1.95	0.00	0.00	0.00
261	piR-hsa-4178299	5.30	4.62	7.23	1.67	1.35	0.00	0.00	0.00	0.00
262	piR-hsa-5411637	2.65	6.15	2.41	0.84	4.05	3.89	0.00	0.00	0.00
263	piR-hsa-1883893	0.00	7.69	0.00	5.86	0.00	3.89	0.00	1.81	0.70
264	piR-hsa-2742244	1.59	0.00	0.00	0.00	4.05	0.00	9.62	1.81	2.79
265	piR-hsa-669874	0.53	0.00	0.00	19.24	0.00	0.00	0.00	0.00	0.00
266	piR-hsa-645846	3.18	4.62	3.61	0.84	1.35	5.84	0.00	0.00	0.00
267	piR-hsa-3776081	0.53	1.54	0.00	9.20	8.09	0.00	0.00	0.00	0.00
268	piR-hsa-211123	2.12	0.00	1.20	5.02	5.39	3.89	0.00	0.90	0.70
269	piR-hsa-2248086	0.53	4.62	6.02	3.35	2.70	1.95	0.00	0.00	0.00
270	piR-hsa-1909905	0.53	0.00	0.00	1.67	1.35	15.57	0.00	0.00	0.00
271	piR-hsa-1593307	3.18	4.62	6.02	1.67	1.35	1.95	0.00	0.00	0.00
272	piR-hsa-2042088	0.00	0.00	0.00	0.00	0.00	0.00	12.83	4.52	1.39
273	piR-hsa-2490509	0.00	1.54	0.00	0.00	1.35	0.00	12.83	0.90	2.09
274	piR-hsa-144277_2	0.00	0.00	0.00	0.00	5.39	0.00	11.23	1.36	0.70
275	piR-hsa-4131663	0.00	0.00	0.00	4.18	4.05	1.95	8.02	0.45	0.00
276	piR-hsa-2542835	1.59	0.00	1.20	0.00	2.70	0.00	11.23	0.45	1.39
277	piR-hsa-1229611	2.12	1.54	1.20	4.18	2.70	5.84	0.00	0.90	0.00
278	piR-hsa-2423519	1.06	0.00	0.00	0.00	4.05	11.68	1.60	0.00	0.00
279	piR-hsa-3021684	4.24	1.54	3.61	7.53	1.35	0.00	0.00	0.00	0.00
280	piR-hsa-2490287	0.00	1.54	0.00	3.35	1.35	1.95	6.42	2.26	1.39
281	piR-hsa-1259933	1.06	1.54	0.00	8.37	2.70	0.00	1.60	2.26	0.70
282	piR-hsa-1302552	0.53	0.00	0.00	5.86	6.74	0.00	0.00	0.00	4.88
283	piR-hsa-67957	1.06	13.85	0.00	0.00	0.00	0.00	0.00	0.90	2.09
284	piR-hsa-1726249	6.89	1.54	7.23	0.84	1.35	0.00	0.00	0.00	0.00
285	piR-hsa-1409954	1.59	1.54	8.43	0.84	5.39	0.00	0.00	0.00	0.00
286	piR-hsa-4053516	2.65	1.54	7.23	1.67	2.70	1.95	0.00	0.00	0.00
287	piR-hsa-2425220	0.00	1.54	1.20	5.02	0.00	0.00	3.21	1.81	4.88
288	piR-hsa-1686806	2.12	3.08	3.61	4.18	2.70	1.95	0.00	0.00	0.00
289	piR-hsa-2319750	2.65	0.00	7.23	1.67	4.05	1.95	0.00	0.00	0.00
290	piR-hsa-4397384	0.00	0.00	3.61	4.18	0.00	9.73	0.00	0.00	0.00
291	piR-hsa-2829413	1.06	0.00	1.20	0.00	1.35	0.00	8.02	0.90	4.88
292	piR-hsa-3839126	0.00	0.00	0.00	7.53	4.05	5.84	0.00	0.00	0.00
293	piR-hsa-3944431	3.71	3.08	3.61	1.67	1.35	3.89	0.00	0.00	0.00
294	piR-hsa-363100_2	2.65	1.54	0.00	1.67	4.05	0.00	3.21	1.36	2.79
295	piR-hsa-6482184	0.53	10.77	1.20	0.84	0.00	3.89	0.00	0.00	0.00

	piRNA	psc1	psc2	psc3	mpc1	mpc2	mpc3	cpc1	cpc2	cpc3
296	piR-hsa-642866	0.00	0.00	0.00	0.00	0.00	0.00	12.83	2.26	2.09
297	piR-hsa-1922210	0.53	0.00	1.20	9.20	0.00	3.89	0.00	0.90	1.39
298	piR-hsa-2436454	3.18	3.08	4.82	1.67	0.00	3.89	0.00	0.45	0.00
299	piR-hsa-7821967_3	0.00	0.00	1.20	0.00	0.00	0.00	11.23	3.16	1.39
300	piR-hsa-4424378	0.00	0.00	0.00	0.00	1.35	0.00	12.83	1.36	1.39
301	piR-hsa-237221	1.59	1.54	0.00	10.87	2.70	0.00	0.00	0.00	0.00
302	piR-hsa-7833890	1.59	1.54	0.00	5.02	2.70	5.84	0.00	0.00	0.00
303	piR-hsa-1905329	0.00	3.08	0.00	2.51	1.35	9.73	0.00	0.00	0.00
304	piR-hsa-4450044	0.53	1.54	1.20	13.38	0.00	0.00	0.00	0.00	0.00
305	piR-hsa-3741185	1.59	0.00	0.00	0.00	1.35	1.95	8.02	0.90	2.79
306	piR-hsa-4144265	1.59	3.08	6.02	0.84	0.00	3.89	0.00	0.45	0.70
307	piR-hsa-4198101	1.59	7.69	1.20	0.84	1.35	3.89	0.00	0.00	0.00
308	piR-hsa-8117137	0.00	0.00	2.41	0.84	0.00	1.95	0.00	0.90	10.46
309	piR-hsa-4100164	1.06	4.62	4.82	1.67	2.70	0.00	1.60	0.00	0.00
310	piR-hsa-3280518	6.36	3.08	3.61	3.35	0.00	0.00	0.00	0.00	0.00
311	piR-hsa-3710717	0.00	0.00	0.00	3.35	1.35	11.68	0.00	0.00	0.00
312	piR-hsa-848451	0.00	0.00	0.00	2.51	4.05	9.73	0.00	0.00	0.00
313	piR-hsa-1057272	0.00	0.00	0.00	0.00	1.35	0.00	11.23	2.26	1.39
314	piR-hsa-343616	0.00	0.00	0.00	2.51	0.00	5.84	1.60	1.36	4.88
315	piR-hsa-2832439	1.59	1.54	1.20	0.84	0.00	1.95	4.81	1.36	2.79
316	piR-hsa-2889978	1.06	1.54	1.20	5.02	1.35	5.84	0.00	0.00	0.00
317	piR-hsa-2529368	3.18	1.54	1.20	0.00	0.00	0.00	1.60	1.36	6.97
318	piR-hsa-3161050	0.00	0.00	0.00	5.86	4.05	3.89	1.60	0.45	0.00
319	piR-hsa-1688824	2.12	1.54	1.20	5.86	2.70	1.95	0.00	0.45	0.00
320	piR-hsa-3638679	4.24	4.62	0.00	1.67	1.35	3.89	0.00	0.00	0.00
321	piR-hsa-2281305	0.00	0.00	1.20	0.00	0.00	0.00	9.62	1.36	3.49
322	piR-hsa-2427082	3.18	4.62	4.82	1.67	1.35	0.00	0.00	0.00	0.00
323	piR-hsa-5062317	0.00	4.62	0.00	0.84	0.00	0.00	0.00	1.81	8.37
324	piR-hsa-1555218	1.59	4.62	6.02	3.35	0.00	0.00	0.00	0.00	0.00
325	piR-hsa-4391981_9	0.00	0.00	0.00	3.35	0.00	3.89	1.60	3.16	3.49
326	piR-hsa-4403628	4.77	3.08	0.00	0.84	0.00	0.00	4.81	0.45	1.39
327	piR-hsa-153942	1.06	1.54	1.20	0.00	0.00	0.00	8.02	1.36	2.09
328	piR-hsa-2856544	4.77	1.54	2.41	1.67	0.00	0.00	3.21	0.90	0.70
329	piR-hsa-1894768	1.59	1.54	1.20	0.00	0.00	1.95	3.21	3.61	2.09
330	piR-hsa-2495779	2.65	1.54	1.20	0.00	4.05	0.00	1.60	2.71	1.39
331	piR-hsa-3706918	0.00	0.00	0.00	3.35	0.00	0.00	6.42	1.81	3.49
332	piR-hsa-4157592	2.12	1.54	1.20	4.18	4.05	1.95	0.00	0.00	0.00
333	piR-hsa-4175186	0.53	1.54	9.63	0.00	1.35	1.95	0.00	0.00	0.00
334	piR-hsa-3714350	0.53	0.00	0.00	9.20	1.35	0.00	3.21	0.00	0.70
335	piR-hsa-354004	1.06	10.77	1.20	0.00	0.00	1.95	0.00	0.00	0.00
336	piR-hsa-3694141	0.00	1.54	1.20	10.87	1.35	0.00	0.00	0.00	0.00
337	piR-hsa-4403262	0.00	0.00	0.00	0.00	0.00	0.00	12.83	0.00	2.09
338	piR-hsa-3383102	1.59	3.08	0.00	4.18	4.05	1.95	0.00	0.00	0.00
339	piR-hsa-3357365	3.71	7.69	1.20	0.84	1.35	0.00	0.00	0.00	0.00
340	piR-hsa-213829	0.53	4.62	1.20	8.37	0.00	0.00	0.00	0.00	0.00
341	piR-hsa-7893387	0.53	0.00	0.00	2.51	8.09	1.95	1.60	0.00	0.00
342	piR-hsa-235205	0.00	0.00	0.00	0.00	0.00	0.00	12.83	0.45	1.39
343	piR-hsa-1416366	0.00	0.00	0.00	11.71	1.35	0.00	1.60	0.00	0.00
344	piR-hsa-1998991	2.12	9.23	0.00	0.00	1.35	1.95	0.00	0.00	0.00
345	piR-hsa-5124632	1.59	7.69	1.20	0.84	1.35	1.95	0.00	0.00	0.00
346	piR-hsa-3365644	0.00	0.00	2.41	7.53	2.70	1.95	0.00	0.00	0.00
347	piR-hsa-1886018	0.53	0.00	0.00	0.00	0.00	0.00	11.23	1.36	1.39
348	piR-hsa-3657078	6.89	0.00	2.41	0.84	1.35	0.00	0.00	0.90	2.09
349	piR-hsa-3104125	4.24	1.54	4.82	2.51	1.35	0.00	0.00	0.00	0.00
350	piR-hsa-4378137_4	0.53	1.54	0.00	4.18	2.70	3.89	0.00	0.90	0.70
351	piR-hsa-1592257	0.00	0.00	0.00	0.00	0.00	0.00	11.23	1.81	1.39
352	piR-hsa-3818788	4.24	0.00	6.02	0.00	4.05	0.00	0.00	0.00	0.00
353	piR-hsa-2839864	1.06	0.00	0.00	0.84	0.00	0.00	6.42	3.16	2.79
354	piR-hsa-1678085	0.00	0.00	1.20	1.67	9.44	1.95	0.00	0.00	0.00

	piRNA	psc1	psc2	psc3	mpc1	mpc2	mpc3	cpc1	cpc2	cpc3
355	piR-hsa-4193743	0.00	0.00	0.00	0.00	0.00	0.00	11.23	0.90	2.09
356	piR-hsa-2537452	0.00	0.00	0.00	14.22	0.00	0.00	0.00	0.00	0.00
357	piR-hsa-4507261	0.00	1.54	0.00	0.00	0.00	0.00	0.00	2.71	9.76
358	piR-hsa-1263612	6.89	0.00	4.82	0.00	0.00	0.00	0.00	0.90	1.39
359	piR-hsa-778924	0.53	1.54	0.00	7.53	2.70	0.00	1.60	0.00	0.00
360	piR-hsa-2165649	1.06	4.62	4.82	0.00	1.35	1.95	0.00	0.00	0.00
361	piR-hsa-1274138	1.06	0.00	0.00	0.00	1.35	1.95	6.42	0.90	2.09
362	piR-hsa-4111185	1.06	3.08	1.20	7.53	0.00	0.00	0.00	0.00	0.70
363	piR-hsa-3693411	0.00	0.00	0.00	5.86	2.70	1.95	1.60	0.00	1.39
364	piR-hsa-1917013	0.53	3.08	0.00	0.00	0.00	0.00	6.42	2.71	0.70
365	piR-hsa-4467055_8	0.00	0.00	2.41	5.86	0.00	3.89	0.00	0.45	0.70
366	piR-hsa-4402141	0.00	0.00	0.00	0.00	0.00	0.00	11.23	1.36	0.70
367	piR-hsa-942735	0.00	0.00	0.00	0.00	0.00	0.00	11.23	1.36	0.70
368	piR-hsa-1574545	0.00	0.00	0.00	0.84	0.00	0.00	9.62	0.00	2.79
369	piR-hsa-2161668	0.00	0.00	1.20	4.18	5.39	1.95	0.00	0.45	0.00
370	piR-hsa-771001	0.53	0.00	0.00	9.20	1.35	0.00	1.60	0.45	0.00
371	piR-hsa-2502273	3.71	0.00	4.82	2.51	0.00	1.95	0.00	0.00	0.00
372	piR-hsa-77303	2.65	1.54	6.02	0.00	1.35	0.00	0.00	0.00	1.39
373	piR-hsa-2853562	0.00	0.00	1.20	8.37	1.35	1.95	0.00	0.00	0.00
374	piR-hsa-2851130	1.59	0.00	0.00	0.00	0.00	0.00	4.81	2.26	4.18
375	piR-hsa-2538984	7.42	0.00	0.00	0.84	1.35	0.00	1.60	0.90	0.70
376	piR-hsa-748358_5	0.00	0.00	0.00	0.00	0.00	0.00	9.62	3.16	0.00
377	piR-hsa-2400882	0.53	0.00	0.00	2.51	5.39	3.89	0.00	0.45	0.00
378	piR-hsa-3610092	5.30	1.54	2.41	0.00	1.35	1.95	0.00	0.00	0.00
379	piR-hsa-727158	5.83	1.54	3.61	0.84	0.00	0.00	0.00	0.00	0.70
380	piR-hsa-1530800	0.00	0.00	0.00	5.86	2.70	3.89	0.00	0.00	0.00
381	piR-hsa-1603046	0.00	0.00	0.00	2.51	4.05	5.84	0.00	0.00	0.00
382	piR-hsa-2518229	0.00	1.54	0.00	0.00	1.35	0.00	3.21	1.81	4.18
383	piR-hsa-3642754	4.77	1.54	4.82	0.84	0.00	0.00	0.00	0.00	0.00
384	piR-hsa-5039329_2	0.00	0.00	0.00	0.00	0.00	0.00	9.62	0.90	1.39
385	piR-hsa-4120912	0.53	0.00	0.00	3.35	5.39	1.95	0.00	0.00	0.70
386	piR-hsa-3263398	5.83	3.08	0.00	0.00	0.00	0.00	0.00	2.26	0.70
387	piR-hsa-2484024	0.00	0.00	0.00	0.84	0.00	0.00	8.02	0.90	2.09
388	piR-hsa-4388882	0.53	1.54	0.00	8.37	1.35	0.00	0.00	0.00	0.00
389	piR-hsa-2499630	0.53	0.00	0.00	0.00	0.00	0.00	8.02	0.90	2.09
390	piR-hsa-2515298	4.24	1.54	0.00	0.00	0.00	0.00	3.21	0.45	2.09
391	piR-hsa-65029	2.12	1.54	4.82	1.67	1.35	0.00	0.00	0.00	0.00
392	piR-hsa-271367	3.71	3.08	2.41	0.84	1.35	0.00	0.00	0.00	0.00
393	piR-hsa-2501382	1.06	0.00	1.20	0.84	0.00	0.00	3.21	2.26	2.79
394	piR-hsa-3345389	0.00	0.00	0.00	3.35	4.05	3.89	0.00	0.00	0.00
395	piR-hsa-1881756	0.00	0.00	0.00	0.00	1.35	0.00	6.42	0.00	3.49
396	piR-hsa-1593686	3.71	3.08	1.20	0.84	0.00	1.95	0.00	0.45	0.00
397	piR-hsa-4359698	2.12	4.62	2.41	0.00	0.00	1.95	0.00	0.00	0.00
398	piR-hsa-1866304	3.18	3.08	4.82	0.00	0.00	0.00	0.00	0.00	0.00
399	piR-hsa-1550295	2.12	7.69	1.20	0.00	0.00	0.00	0.00	0.00	0.00
400	piR-hsa-550050	0.00	0.00	0.00	0.00	0.00	0.00	6.42	3.16	1.39
401	piR-hsa-76694	3.71	0.00	7.23	0.00	0.00	0.00	0.00	0.00	0.00
402	piR-hsa-2491463	4.24	3.08	0.00	0.00	0.00	1.95	0.00	0.90	0.70
403	piR-hsa-2534860	1.06	0.00	0.00	0.00	1.35	3.89	0.00	3.16	1.39
404	piR-hsa-2715002	0.00	0.00	0.00	0.00	0.00	0.00	8.02	1.36	1.39
405	piR-hsa-2511205	0.53	0.00	0.00	0.84	0.00	0.00	3.21	4.07	2.09
406	piR-hsa-4271500	0.53	1.54	0.00	6.69	0.00	1.95	0.00	0.00	0.00
407	piR-hsa-4150185	1.06	0.00	1.20	8.37	0.00	0.00	0.00	0.00	0.00
408	piR-hsa-1943369	0.00	0.00	0.00	5.02	5.39	0.00	0.00	0.00	0.00
409	piR-hsa-2447480	1.59	3.08	4.82	0.84	0.00	0.00	0.00	0.00	0.00
410	piR-hsa-1416960	2.65	4.62	1.20	1.67	0.00	0.00	0.00	0.00	0.00
411	piR-hsa-699024	0.00	0.00	1.20	0.00	0.00	0.00	6.42	1.81	0.70
412	piR-hsa-6764147	0.00	0.00	1.20	0.84	0.00	0.00	0.00	1.81	6.28
413	piR-hsa-644441_3	0.53	0.00	0.00	0.00	0.00	0.00	6.42	3.16	0.00

	piRNA	psc1	psc2	psc3	mpc1	mpc2	mpc3	cpc1	cpc2	cpc3
414	piR-hsa-2500917	0.53	0.00	1.20	0.00	0.00	0.00	4.81	1.36	2.09
415	piR-hsa-1160813	0.00	0.00	0.00	6.69	1.35	1.95	0.00	0.00	0.00
416	piR-hsa-628215	0.00	0.00	0.00	0.00	0.00	0.00	6.42	0.00	3.49
417	piR-hsa-5313694	0.00	0.00	0.00	0.00	0.00	0.00	4.81	4.97	0.00
418	piR-hsa-778446	0.53	0.00	0.00	0.00	0.00	0.00	1.60	1.36	6.28
419	piR-hsa-313568	0.00	0.00	0.00	8.37	1.35	0.00	0.00	0.00	0.00
420	piR-hsa-3153428	2.65	4.62	2.41	0.00	0.00	0.00	0.00	0.00	0.00
421	piR-hsa-2365951	0.00	0.00	0.00	5.86	1.35	1.95	0.00	0.45	0.00
422	piR-hsa-4398177	0.00	0.00	0.00	8.37	0.00	0.00	0.00	0.45	0.70
423	piR-hsa-5884872	1.59	3.08	4.82	0.00	0.00	0.00	0.00	0.00	0.00
424	piR-hsa-851842	3.71	0.00	3.61	0.00	0.00	1.95	0.00	0.00	0.00
425	piR-hsa-4175241	0.53	0.00	1.20	7.53	0.00	0.00	0.00	0.00	0.00
426	piR-hsa-612387	3.71	3.08	2.41	0.00	0.00	0.00	0.00	0.00	0.00
427	piR-hsa-1551388	0.00	0.00	0.00	0.00	0.00	0.00	4.81	2.26	2.09
428	piR-hsa-4049552	0.00	0.00	0.00	0.00	1.35	0.00	3.21	1.81	2.79
429	piR-hsa-42060	1.06	0.00	0.00	6.69	1.35	0.00	0.00	0.00	0.00
430	piR-hsa-338024	3.18	1.54	2.41	0.00	0.00	0.00	0.00	0.45	1.39
431	piR-hsa-2030201	3.71	1.54	3.61	0.00	0.00	0.00	0.00	0.00	0.00
432	piR-hsa-4135859	0.00	0.00	0.00	0.84	0.00	0.00	3.21	4.07	0.70
433	piR-hsa-1692624	0.00	0.00	0.00	6.69	0.00	1.95	0.00	0.00	0.00
434	piR-hsa-1534285	2.12	1.54	4.82	0.00	0.00	0.00	0.00	0.00	0.00
435	piR-hsa-2832794	0.00	0.00	0.00	0.00	0.00	0.00	4.81	2.26	1.39
436	piR-hsa-1370047	0.00	0.00	0.00	0.84	0.00	0.00	0.00	5.87	1.39
437	piR-hsa-632933	0.00	0.00	0.00	0.00	1.35	0.00	1.60	2.26	2.79
438	piR-hsa-2069180	4.24	0.00	1.20	0.84	1.35	0.00	0.00	0.00	0.00
439	piR-hsa-1524373	0.00	0.00	0.00	0.00	0.00	0.00	1.60	0.90	4.88
440	piR-hsa-2487038_7	0.00	0.00	0.00	0.00	0.00	0.00	0.00	1.81	5.58
441	piR-hsa-5568482	0.00	0.00	0.00	0.00	0.00	0.00	0.00	3.61	3.49
442	piR-hsa-4346643	0.00	0.00	0.00	0.84	0.00	0.00	0.00	4.07	2.09
443	piR-hsa-7313613	0.53	1.54	0.00	0.00	0.00	0.00	0.00	3.61	0.70
444	piR-hsa-4398796	4.77	0.00	0.00	0.00	0.00	0.00	0.00	1.36	0.00
445	piR-hsa-2369565	0.00	0.00	0.00	0.00	0.00	0.00	0.00	0.90	4.88
446	piR-hsa-7098338	0.00	0.00	0.00	0.00	0.00	0.00	1.60	3.16	0.70
447	piR-hsa-7670531_2	0.00	0.00	0.00	0.00	0.00	0.00	0.00	3.61	1.39
